# Single nucleotide polymorphisms in asthma candidate genes *TBXA2R*, *ADAM33 FCER1B* and *ORMDL3* in Pakistani asthmatics a case control study

**DOI:** 10.1186/s40733-018-0039-4

**Published:** 2018-03-22

**Authors:** Nusrat Saba, Osman Yusuf, Sadia Rehman, Saeeda Munir, Amna Noor, Muhammad Saqlain, Atika Mansoor, Ghazala Kaukab Raja

**Affiliations:** 1 Institute of Biomedical and Genetic Engineering, G-9/1, Islamabad, Pakistan; 2 The Allergy and Asthma Institute of Pakistan, 275, Gomal Road, Islamabad, E-7 Pakistan; 3 Department of Biochemistry, Pir Mehar Ali Shah Arid Agriculture University Rawalpindi, Rawalpindi, Pakistan; 4 Rawalpindi Medical College, Rawalpindi, Pakistan

**Keywords:** Asthma, Genetic polymorphisms, Pakistan, Genetic markers

## Abstract

**Background:**

Genetic variations in different loci and genes are important in asthma pathogenesis. There is much importance of various immunological pathways in the IgE secretion regulation. Alterations in any main part of these pathways can increase the risk of asthma development. Polymorphisms in these genetic markers can effect certain pathways which predict the asthma susceptibility. In the present study, SNPs directly or indirectly affecting the immunological process pathways are selected.

**Methods:**

This study was conducted to determine association of 16 SNPs in 10 candidate genes with asthma in Pakistani population in 333 asthmatic cases and 220 healthy controls. Genotyping was performed using the Sequenom Mass ARRAY iPLEX platform (14 SNPs) and TaqMan assay (2 SNPs).

**Results:**

The minor allele at two of the SNPs showed association with protection from asthma, rs1131882 in *TBXA2R* gene (OR 0.73, 95% CI 0.52–1.01, *P* = 0.05) and rs2280091 in the *ADAM33* gene (OR 0.69, 95% CI 0.50–0.97, *P* = 0.03). For *FCER1B* gene, rs2583476 the asthmatic male gender had higher TT genotype counts as compared to controls (OR = 1.86, 95% CI = 1.09–3.17, *p* = 0.01). In rs11650680 of *ORMDL3* gene the CT genotype is more prevalent in female asthma cases in comparison with female controls (OR = 1.99, 95% CI = 1.02–3.89, *p* = 0.03).

**Conclusions:**

This data suggests that variations at *TBXA2R* and *ADAM33* genes are found to be associated with asthma susceptibility in Pakistan. *FCER1B* gene is associated with male and *ORMDL3* in female asthmatics. These genetic markers can be important source of asthma risk in Pakistani population.

**Electronic supplementary material:**

The online version of this article (10.1186/s40733-018-0039-4) contains supplementary material, which is available to authorized users.

## Background

Asthma is a disease of the lower airways that is remarkably heterogeneous between affected individuals [1]. According to defination of GINA (Global Initiative for Asthma) "asthma is a disorder defined by its clinical, physiological and pathological characteristics. Asthma is the chronic (long-lasting) inflammatory disease of the airways in which many cells and cellular pathways are involved". In those susceptible to asthma, this inflammation causes the airways to spasm and swell periodically so that the airways narrow. The individual then must wheeze or gasp for air. Obstruction to air flow either resolves spontaneously or responds to a wide range of treatments, but continuing inflammation makes the airways hyper-responsive to stimuli such as cold air, exercise, dust mites, pollutants in the air, and even stress and anxiety.

According to recent report of GINA "Asthma is the problem worldwide with an estimated 300 million affected individuals. Nonetheless, based on the standardized methods for assessing asthma symptoms, it appears that global prevalence of asthma ranges from 1-16% of the population in different countries". Social and economical factors are integral to understanding asthma and its care, from the perspective of both the individual person with asthma and the healthcare provider. The costs of asthma depends on its prevalence, the individual patient’s level of asthma control, the extent to which exacerbations are avoided, and the cost of medical care and medications. According to the latest WHO data published in may 2014 Asthma Deaths in Pakistan reached 10,817 or 0.96% of total deaths. The age adjusted Death Rate is 10.30 per 100,000 of population ranks Pakistan number 41 in the world.

Many biological pathways and genes in those pathways have been implicated in asthma pathogenesis. Variants in over 100 genes have been associated with asthma, but all with small individual effect sizes. It is likely that many genes act in concert to determine individual-specific risks for asthma [2]. The genetic factors add approximately 79% alone in asthma pathogenesis [3]. The causes of asthma are not fully understood. Both genetic and environmental factors are involved, but how these factors interact to confer risk is still largely unknown. We selected 16 single nucleotide polymorphisms (SNPs) in 10 genes for genotyping in Pakistani asthma cases and controls.

## Methods

### Patient population and study design

The ethical review committee of the parent organization approved this project (ERC-08-01). Written informed consent was obtained from all participants. Asthmatic subjects were recruited from Islamabad and Lahore. The final sample included 333 Pakistani adult subjects with an asthma diagnosis provided by a pulmonologist. Two hundred non-asthmatic healthy controls were recruited from the general population to be similar to the cases with respect to ethnicity and proportions of males and females.

### Blood sample collection, DNA extraction, and genotyping

A venous blood sample was obtained from each study participant, and genomic DNA was extracted from whole blood using a standard phenol chloroform extraction protocol [4]. Fourteen SNPs were genotyped using a Sequenom iPLEX assay and two SNPs were genotyped using TaqMan assays and analyzed on an ABI 7900 HT Fast Real Time PCR (Applied Biosystems, USA). All genotyping was performed at the University of Chicago USA.

### SNP and gene selection

We selected SNPs in genes that are involved and implicated in asthma risk. Genes involved in immunological and allergic pathways are important in asthma pathogenesis. Therefore, based on results of previous candidate gene and genome-wide studies, we selected 16 single nucleotide polymorphisms (SNPs) in 10 genes for genotyping in Pakistani asthma cases and controls. The selection of genes is purely rely on the immunological and allergic pathways and have been reported in some populations to be strongly associated with asthma. These SNPs from these genes are selected as these are found to have strong effect in either pathogenesis or protection of asthma in some of closely related populations. And we want to check that this is the same result as previously reported or our population has different association.

### Quality checks and statistical analyses

Hardy-Weinberg equilibrium (HWE) was determined in the entire sample and separately in the cases and controls all the SNPs were in Hardy-Weinberg equilibrium. Multiple logistic regression was used to estimate odds ratios (ORs) and 95% confidence intervals (CIs) for each SNP. Linkage disequilibrium (LD) between the 16 SNPs in our sample was determined using matSpD program: (http://gump.qimr.edu.au/general/daleN/matSpD/). There was no LD between SNPs. Therefore all tests were considered to be independent.

## Results

### Demographic and genotyping characteristics

The asthma cases included 148 (44.5%) males and 185 (55.5%) females; the mean age was 40 ± SE = 0.93 years. The controls included 88 males (44.0%) males and 112 (56.0%)) females with mean age of 30 ± SE = 0.97 years. The genotyping methods, call rates, minor allele frequencies, and Hardy-Weinberg *p*-value calculated as a group are shown for all 16 SNPs in Table 1.

**Table 1:** Single Nucleotide Polymorphisms (SNPs) genotyped in asthma patients

GENE	Chr location	rs #	Literature cited	Genotyping technique	Call Rate	Allele (minor/major)	MAF	HWE p-value
*ADRB2*	5	rs1042713	[19]	iPLEX	0.962	A/G	0.432	0.566
*HLA-G*	6	rs1063320	[20]	Taqman	0.993	C/G	0.257	0.090
*NOS3*	7	rs1799983	[21]	iPLEX	0.969	T/G	0.185	0.655
*NOS3*	7	rs1800779	[22]	iPLEX	0.964	G/A	0.197	0.233
*FCER1B*	11	rs2583476	[23]	iPLEX	0.966	C/T	0.480	0.191
*NOS1*	12	rs2682826	[24]	iPLEX	0.968	T/C	0.292	0.327
*ORMDL3*	17	rs11650680	[25, 26]	iPLEX	0.969	T/C	0.220	0.602
*GSDMA*	17	rs3894194	[27]	iPLEX	0.955	T/C	0.491	0.767
*ORMDL3*	17	rs7216389	[26]	Taqman	0.901	C/T	0.413	0.199
*ORMDL3*	17	rs8079416	[27]	iPLEX	0.958	T/C	0.486	0.775
*TBXA2R*	19	rs1131882	[28]	iPLEX	0.964	A/G	0.188	0.247
*TFGB1* ^*a*^	19	rs1800469	[29]	iPLEX	0.957	T/C	0.353	0.022
*TBXA2R*	19	rs4523	[30, 31]	iPLEX	0.955	C/T	0.480	0.380
*ADAM33*	20	rs2280091	[32]	iPLEX	0.944	G/A	0.192	0.060
*ADAM33*	20	rs528557	[11, 33]	iPLEX	0.908	G/C	0.393	0.163
*ADAM33*	20	rs543749	[11]	iPLEX	0.940	T/G	0.189	0.334

*MAF* minor allele frequency, *HWE* Hardy-Weinberg equilibrium

^a^SNP not in HWE so excluded from further analysis

### Allelic and genotypic associations

Four SNPs showed evidence for an association with asthma at a *p* < 0.05. Results of all analyses are shown in Additional file 1: Table S1.

The minor alleles at SNPs in two genes, *TBXA2R* and *ADAM33*, were more frequent in the controls compared to the asthmatics. The odds ratio (OR) for the minor (A) allele at rs1131882 in *TBXA2R* was 0.73 (95% CI 0.52–1.01). There were more AA homozygotes in the controls compared to cases. The OR for the minor (G) allele at rs2280091 in *ADAM33* G was 0.69 (95% CI 0.5–0.97). There were more GG homozygotes in controls.

### Gender based association between SNPs and asthma

All age and gender adjusted genotyped data was also explored for gender based disease association. Two of the SNPs showed gender based associations with asthma (Table 2). *FCER1B* gene SNP rs2583476 showed a significant genotype distribution in males. The homozygous TT and CC genotype frequencies in cases were; TT 37.5% and CC 24.3% as compared with controls; TT 24% and CC 27.7% respectively. The asthmatic males had higher TT genotype counts as compared to controls (OR = 1.99, 95% CI = 1.02–3.89, *p* = 0.03). In females, a significant association was observed in rs11650680 (*ORMDL3)*. In case-control data, the CT genotype frequency in female cases is 36% and 23% in control female subjects. The CT genotype is more prevalent in female asthmatics as compared to female control group (OR = 1.86, 95% CI = 1.09–3.17, *p* = 0.01).

**Table 2:** Genotype distribution significant among male and female gender in cases and controls and Odds Ratio with *p* values

SNP	Gene	Genotype	Female			Male			*p*-value
			Cases	Controls	OR(95%CI)	Cases	Controls	OR(95%CI)	
rs2583476	*FCER1B*	T/T	42	34	1	54	22	**1.99 (1.02–3.89)**	
		C/T	102	52	1.59 (0.91–2.79)	55	43	1.04 (0.57–1.89)	**0.033**
		C/C	38	32	0.96 (0.5–1.85)	35	25	1.13 (0.57–2.25)	
rs11650680	*ORMDL3*	C/C	106	82	1	91	46	1.53 (0.97–2.42)	
		C/T	65	27	**1.86 (1.09–3.17)**	48	39	0.95 (0.57–1.59)	**0.01**
		T/T	8	7	0.88 (0.31–2.54)	9	3	2.32 (0.61–8.85)	

## Discussion

This candidate gene study was conducted in Pakistani asthma patients and healthy controls. This is the first study to report associations between SNPs in candidate genes and asthma in Pakistani population. In the present study, we investigated the association between 16 SNPs in 10 candidate genes (Additional file 1: Table S1) for asthma in the Pakistani population. All of the genes included in the present study were either directly or indirectly involved in pathways affecting the immunological process. Asthma is caused by interaction of multiple genes, some of which have a protective effect and others contribute to the pathogenesis of the disease, with each gene having its own tendency to be influenced by the environment.

Thromboxane A2 receptor (*TBXA2R*) is a potent broncho- and vasoconstrictor and is associated with leukotriene synthesis. It is involved in prostaglandin and leukotriene pathways, and has diverse physiological and pathophysiological actions related to allergies, modulation of acquired immunity, atherogenesis, and neovascularization [5]. Polymorphisms in the *TBXA2R* gene have been associated with urticaria [6]. This study investigated associations between asthma and a *TBXA2R* polymorphism. We have found an association between rs1131882 (*TBXA2R*) and asthma in Pakistani population. The A allele and AA genotypes are associated with protection in the Pakistani population. In a study of other variants in this gene in Japanese subjects, *TBXA2R* alleles were also associated with asthma-related phenotypes [7]. Thromboxane pathways therefore play important roles in airway inflammation and remodeling in asthma patients.

For all genotyped SNPs, no gender specific disease association was seen except for *FCER1B* gene SNP rs2583476. In this SNP the asthmatic male gender had higher TT genotype counts as compared to controls. It is clearly indicated that in our sample population of male gender TT genotype is highly prevalent in asthma patients as compared to healthy controls, whereas CC genotype is not different between males in cases and controls. Gender specific association with this SNP in asthma patients has not been reported in different world populations and this is reported in our population only. Replication in a larger study of male subjects will clarify differences between Pakistani subjects and those of other populations.

In *ORMDL3* gene SNP rs11650680, a significant association was found with female asthmatics. The heterozygous CT genotype is more prevalent in female asthmatics as compared to female controls. These results clearly show that CT genotype of *ORMDL3* gene SNP rs11650680 is highly prevalent in female asthma cases as compared to healthy controls in our study population. A comparison was also made between male and female subjects with regards to genotype frequencies of studied SNPs under case-control model. For *FCER1B* gene SNP rs2583476 significant difference existed in genotype frequencies among male and female cases (OR = 1.86, 95% CI = 1.09–3.17, *p* = 0.01). The odds ratio results clearly indicate that male subjects are at higher risk of asthma as compared to female counter-parts. Genotype frequencies also demonstrated a high prevalence of risk alleles in males as compared to females. In the case of *ORMDL3* SNP rs11650680, our results indicate that female asthmatic carriers of the CT genotype are at higher risk of disease as compared to male asthmatic subjects (OR = 1.99, 95% CI = 1.02–3.89, *p* = 0.03). This association with gender has not been reported in other populations and this may be due to our population genetic data is some different from other world population. This point has to be explored further in Pakistani population and needs to be taken into consideration and will be helpful in studying asthma pathogenesis in adults. This is a different and unique result we have found in this study.

This may represent an ethnic difference in the genetic makeup of Pakistanis compared to other ethnicity groups. The first strong genetic evidence suggesting that genes in non-allergic, non-immune pathways may play important roles in asthma pathogenesis was the report of *ADAM33* as the first positionally-cloned asthma gene [1]. *ADAM33* encodes a disintegrin and metalloprotein-33 protein that participates in the bronchial remodeling process in asthma [8]. Asthma is a complex disease, but learning more about how SNP mutations in *ADAM33* give rise to asthmatic conditions will provide important clues in treating asthma. The *ADAM33* gene is expressed in lung fibroblasts and bronchial smooth muscle. Strong associations of rs2280091 *(ADAM33)* have been demonstrated in Taiwanese [9], Saudi [10], and an Asian population [11]. We also report a relatively strong association with this SNP (recessive model *p* = 0.005), in which the G allele has a protective effect, in contrast to the above-mentioned studies in which the G allele was associated with risk. This difference may be due to different environmental exposures in our subjects or differences in the frequencies of relevant background genes in the Pakistani population and those in previous studies. It has been speculated that *ADAM33* may have cytokine stimulating effects, given that other *ADAM* proteins (*ADAM10* and *ADAM17*) also appear to interact with inflammatory cytokines [12]. Cysteine-rich and EGF domains of *ADAM33* have been identified to have a role in cell adhesion and membrane fusion events [13]. These properties of *ADAM33* suggest that it might play a role in progression of asthma [14–16].

The lack of a very strong associations in our data could be due to the relatively small size of the study sample and the fact that the subjects in our study were adults whereas most of the previous genetic studies were performed largely in children with asthma [7, 10, 17, 18]. Our findings are important in world genetic pool of asthma cases and will be helpful in further asthma pathogenesis based on these genes. These genes have to be screened fully in asthmatics in further studies instead of few SNPs and this will add information about Pakistani asthmatics in world data. Afterwards, we will be able to tell how Pakistani population genetic makeup is different from other world.

## Conclusions

The present study suggests that variations at *TBXA2R* and *ADAM33* genes are linked with asthma susceptibility in Pakistan. These results are same as found in Caucasians as Pakistani population is closed to it in genetic make up. *FCER1B* gene is associated with male and *ORMDL3* in female asthmatics. Gender based association has not been seen in other studies so this suggest our population genetic data is somewhat different from rest of world. These genetic markers need to be explored fully and will be helpful in studying asthma pathogenesis in adults. These genetic markers can be important source of asthma risk in Pakistani population and needs to be studied in detail with large number of samples.

## Additional file

Additional file 1: **Table S1.** Association of Genotype and Allele Frequencies with Asthma Among Cases and Controls. (DOC 81 kb)

## References

[1] Ober C, Yao TC (2011). The genetics of asthma and allergic disease: a 21^st^ century perspective. Immunol Rev.

[2] Nicolae DL, Ober C. (Too) great expectations: the challenges in replicating asthma disease genes. Am J Respir Crit Care Med 2009 179: 1078–1079. 10.1164/rccm.200903-0456ED PMC4263324 19498061

[3] Holgate ST, Yang Y, Haitchi HM, Powell RM, Holloway JW, Yoshisue H, Pang YY, Cakebread J, Davies DE (2006). The genetics of asthma ADAM33 as an example of a susceptibility gene. Proc Am Thorac Soc.

[4] Sambrook J, MacCallum P, Russell D (2000). Molecular cloning: a laboratory manual.

[5] Nakahata N (2008). Thromboxane A2: physiology/pathophysiology, cellular signal transduction and pharmacology. Pharmacol Ther.

[6] Palikhe NS, Kim SH, Lee HY, Kim JH, Ye YM, Park HS (2011). Association of thromboxane A2 receptor (*TBXA2R*) gene polymorphism in patients with aspirin-intolerant acute urticaria. Clin Exp Allergy.

[7] Takeuchi K, Mashimo Y, Shimojo N, Arima T, Inoue Y, Morita Y (2013). Functional variants in the thromboxane A2 receptor gene are associated with lung function in childhood-onset asthma. Clin Exp Allergy.

[8] Vergaraa CI, Acevedoa N, Jiméneza S, Martíneza B, Mercadoa D, Gusmãoc L (2010). Six- SNP haplotype of *ADAM33* is associated with asthma in a population of Cartagena, Colombia. Int Arch Allergy Immunol.

[9] Chiang CH, Lin MW, Chung MY, Yang UC (2012). The association between the *IL-4*, *ADRβ2* and *ADAM 33* gene polymorphisms and asthma in the Taiwanese population. J Chin Med Assoc.

[10] Al-Khayyat AI, Al-Anazi M, Warsy A, Vazquez-Tello A, Alamri AM, Halwani R (2012). T1 and T2 *ADAM33* single nucleotide polymorphisms and the risk of childhood asthma in a Saudi Arabian population: a pilot study. Ann Saudi Med.

[11] Liang S, Wei X, Gong C, Wei J, Chen Z, Deng JA (2013). Disintegrin and metalloprotease 33 (*ADAM33*) gene polymorphisms and the risk of asthma: a meta-analysis. Hum Immunol.

[12] Redington AE, Roche WR, Holgate ST, Howarth PH (1998). Co-localization of immunoreactive transforming growth factor-beta 1 and decorin in bronchial biopsies from asthmatic and normal subjects. J Pathol.

[13] Iba K, Albrechtsen R, Gilpin B, Fröhlich C, Loechel F, Zolkiewska A (2000). The cysteine-rich domain of human ADAM 12 supports cell adhesion through syndecans and triggers signaling events that lead to beta1 integrin-dependent cell spreading. J Cell Biol.

[14] Kedda MA, Duffy DL, Bradley B, O'Hehir RE, Thompson PJ (2006). ADAM33 haplotypes are associated with asthma in a large Australian population. Eur J Hum Genet.

[15] Holloway JW, Keith TP, Davies DE, Powell R, Haitchi HM, Keith TP, Holgate ST (2004). The discovery and role of *ADAM 33* a new candidate gene for asthma. Expert Rev Mol Med.

[16] Sharma N, Tripathi P, Awasthi S (2011). Role of *ADAM33* gene and associated single nucleotide polymorphisms in asthma. Allergy Rhinol (Providence).

[17] Kang SH, Kim HY, Seo JH, Kwon JW, Jung YH, Song YH (2012). Bronchial hyperresponsiveness to methacholine and AMP in children with atopic asthma. Allergy Asthma Immunol Res.

[18] Hussein YM, Shalaby SM, Mohamed RH, Hassan TH (2011). Association between genes encoding components of the *IL-10/IL-10* receptor pathway and asthma in children. Ann Allergy Asthma Immunol.

[19] Basu K, Palmer CN, Tavendale R, Lipworth BJ, Mukhopadhyay S (2009). Adrenergic beta(2)-receptor genotype predisposes to exacerbations in steroid-treated asthmatic patients taking frequent albuterol or salmeterol. J Allergy Clin Immunol.

[20] Zheng XQ, Li CC, Xu DP, Lin A, Bao WG, Yang GS, Yan WH (2010). Analysis of the plasma soluble human leukocyte antigen-G and interleukin-10 levels in childhood atopic asthma. Hum Immunol.

[21] Coto-Segura P, Coto E, Mas-Vidal A, Morales B, Alvarez V, Díaz M, Alonso B, Santos-Juanes J (2011). Influence of endothelial nitric oxide synthase polymorphisms in psoriasis risk. Arch Dermatol Res.

[22] Kuzmanić Samija R, Primorac D, Resić B, Lozić B, Krzelj V, Tomasović M, Stoini E, Samanović L, Benzon B, Pehlić M, Boraska V, Zemunik T (2011). Association of *NOS3* tag polymorphisms with hypoxic-ischemic encephalopathy. Croat Med J.

[23] Hizawa N, Maeda Y, Konno S, Fukui Y, Takahashi D, Nishimura M (2006). Genetic polymorphisms at *FCER1B* and *PAI-1* and asthma susceptibility. Clin Exp Allergy.

[24] Ibarrola-Villava M, Peña-Chilet M, Fernandez LP, Aviles JA, Mayor M, Martin-Gonzalez M, Gomez-Fernandez C, Casado B, Lazaro P, Lluch A, Benitez J, Lozoya R, Boldo E, Pizarro A, Martinez-Cadenas C (2011). Genetic polymorphisms in DNA repair and oxidative stress pathways associated with malignant melanoma susceptibility. Eur J Cancer.

[25] Li FX, Tan JY, Yang XX, Wu YS, Wu D, Li M (2012). Genetic variants on 17q21 are associated with asthma in a Han Chinese population. Genet Mol Res.

[26] Kang MJ, Yu HS, Seo JH, Kim HY, Jung YH, Kim YJ, Kim HJ, Lee SY, Hong SJ (2012). *GSDMB*/*ORMDL3* variants contribute to asthma susceptibility and eosinophil-mediated bronchial hyperresponsiveness. Hum Immunol.

[27] Marinho S, Custovic A, Marsden P, Smith JA, Simpson A (2012). 17q12-21 variants are associated with asthma and interact with active smoking in an adult population from the United Kingdom. Ann Allergy Asthma Immunol.

[28] Kim JH, Lee SY, Kim HB, Jin HS, Yu JH, Kim BJ, Kim BS, Kang MJ, Jang SO, Hong SJ (2008). *TBXA2R* gene polymorphism and responsiveness to leukotriene receptor antagonist in children with asthma. Clin Exp Allergy.

[29] Yang XX, Li FX, Wu YS, Wu D, Tan JY, Li M (2011). Association of *TGF-beta1*, *IL-4* and *IL-13* gene polymerphisms with asthma in a Chinese population. Asian Pac J Allergy Immunol.

[30] Lee S, Kim J, Kim B, Choi S, Lee S, Hong S (2009). Association between *TBXA2R* T924C polymorphism and pulmonary function in Korean children with asthma. Clin Biochem.

[31] Murk W, Walsh K, Hsu LI, Zhao L, Bracken MB, Dewan AT (2011). Attempted replication of 50 reported asthma risk genes identifies a SNP in *RAD50* as associated with childhood atopic asthma. Hum Hered.

[32] Lee YH, Song GG (2012). Association between *ADAM33* T1 polymorphism and susceptibility to asthma in Asians. Inflamm Res.

[33] Miyake Y, Tanaka K, Arakawa M (2012). *ADAM33* polymorphisms, smoking and asthma in Japanese women: the Kyushu Okinawa Maternal and Child Health Study. Int J Tuberc Lung Dis.

