# Supplementary material for: Single nucleotide polymorphisms in asthma candidate genes TBXA2R, ADAM33 FCER1B and ORMDL3 in Pakistani asthmatics a case control study
Source: Asthma Res Pract. 2018 Mar 22;4:4. doi: 10.1186/s40733-018-0039-4 (PMC5863901; doi:10.1186/s40733-018-0039-4)
Supplement: Supplementary file 1 — Table S1. Association of Genotype and Allele Frequencies with Asthma Among Cases and Controls. (DOC 81 kb) [file 40733_2018_39_MOESM1_ESM.doc]

**Additional file 1: Table S1. Association of Genotype and Allele Frequencies with Asthma Among Cases and Controls**

| **SNP** | **sample** | **Minor Allele** | **Major**  **Allele** | **Homozygotes**  **Minor** | **Heterozygotes** | **Homozygotes**  **Major** | **odds Ratio** | **95%CI** |
| --- | --- | --- | --- | --- | --- | --- | --- | --- |
|  |  | A | G | AA | AG | GG |  |  |
| rs1042713 | case | 284 | 364 | 56 | 172 | 96 | 1.00 | (0.77-1.30) |
|  | control | 150 | 192 | 37 | 76 | 58 |  |  |
|  |  | C | G | CC | CG | GG |  |  |
| rs1063320 | case | 161 | 501 | 25 | 111 | 195 | 0.88 | (0.66-1.18) |
|  | control | 98 | 268 | 16 | 66 | 101 |  |  |
|  |  | T | G | TT | GT | GG |  |  |
| rs1799983 | case | 114 | 540 | 12 | 90 | 225 | 0.86 | (0.61-1.20) |
|  | control | 68 | 276 | 7 | 54 | 111 |  |  |
|  |  | G | A | GG | AG | AA |  |  |
| rs1800779 | case | 132 | 510 | 17 | 98 | 206 | 1.07 | (0.77-1.49) |
|  | control | 68 | 282 | 7 | 54 | 114 |  |  |
|  |  | C | T | CC | CT | TT |  |  |
| rs2583476 | case | 297 | 349 | 70 | 157 | 96 | 0.80 | (0.62-1.04) |
|  | control | 180 | 170 | 48 | 84 | 43 |  |  |
|  |  | T | C | TT | CT | CC |  |  |
| rs2682826 | case | 196 | 456 | 25 | 146 | 155 | 1.08 | (0.81-1.44) |
|  | control | 98 | 246 | 12 | 74 | 86 |  |  |
|  |  | T | C | TT | CT | CC |  |  |
| rs11650680 | case | 146 | 508 | 17 | 112 | 198 | 1.11 | (0.80-1.52) |
|  | control | 71 | 273 | 8 | 55 | 109 |  |  |
|  |  | T | C | TT | CT | CC |  |  |
| rs3894194 | case | 317 | 329 | 77 | 163 | 83 | 0.94 | (0.72-1.22) |
|  | control | 171 | 167 | 48 | 75 | 46 |  |  |
|  |  | C | T | CC | CT | TT |  |  |
| rs7216389 | case | 244 | 368 | 51 | 142 | 113 | 0.91 | (0.69-1.19) |
|  | control | 137 | 187 | 33 | 71 | 58 |  |  |
|  |  | T | C | TT | CT | CC |  |  |
| rs8079416 | case | 310 | 338 | 73 | 164 | 87 | 0.91 | (0.70-1.18) |
|  | control | 171 | 169 | 48 | 75 | 47 |  |  |
|  |  | A | G | AA | AG | GG |  |  |
| rs1131882 | case | 110 | 540 | 7 | 96 | 222 | 0.73 | (0.52-1.01) |
|  | control | 75 | 267 | 13 | 49 | 109 |  |  |
|  |  | C | T | CC | CT | TT |  |  |
| rs4523 | case | 301 | 335 | 71 | 159 | 88 | 0.95 | (0.73-1.24) |
|  | control | 170 | 180 | 46 | 78 | 51 |  |  |
|  |  | G | A | GG | AG | AA |  |  |
| rs2280091 | case | 105 | 531 | 9 | 87 | 222 | 0.69 | (0.50-0.97) |
|  | control | 75 | 263 | 15 | 45 | 109 |  |  |
|  |  | G | C | GG | CG | CC |  |  |
| rs528557 | case | 232 | 368 | 53 | 126 | 121 | 1.00 | (0.76-1.32) |
|  | control | 129 | 205 | 25 | 79 | 63 |  |  |
|  |  | T | G | TT | GT | GG |  |  |
| rs543749 | case | 128 | 508 | 13 | 102 | 203 | 1.17 | (0.83-1.65) |
|  | control | 59 | 275 | 8 | 43 | 116 |  |  |
